# Self‐esteem as an important factor in quality of life and depressive symptoms in anosmia: A pilot study

**DOI:** 10.1111/coa.12855

**Published:** 2017-03-23

**Authors:** K. Kollndorfer, J.L. Reichert, B. Brückler, V. Hinterleitner, V. Schöpf

**Affiliations:** ^1^ Department of Biomedical Imaging and Image‐guided Therapy Medical University of Vienna Vienna Austria; ^2^ Institute of Psychology University of Graz Graz Austria; ^3^ BioTechMed Graz Austria

**Keywords:** olfaction, olfactory dysfunction, quality of life, self‐esteem, sensory loss

## Abstract

**Objectives:**

Previous research has reported a negative impact of olfactory dysfunction on quality of life (QoL) and depressive symptoms. As self‐esteem was identified as a contributing factor to depression, this study aimed to investigate QoL, depressive symptoms and self‐esteem in patients with smell loss.

**Design:**

Prospective controlled study.

**Setting:**

Department of Biomedical Imaging and Image‐guided Therapy, Medical University of Vienna, in co‐operation with the Department of Ear, Nose and Throat Diseases, Medical University of Vienna, Austria.

**Participants:**

Twenty‐two anosmic patients (12 females, 10 males) and 25 healthy controls (15 females, 10 males) participated in this study.

**Main outcome measures:**

Olfactory performance was assessed using the Sniffin’ Sticks battery. In addition, psychological questionnaires that covered the topics quality of life (WHOQOL‐BREF), depressive symptoms (BDI‐II) and self‐esteem (MSWS) were conducted.

**Results:**

The results of this study revealed a decrease in QoL and reduced body‐related self‐esteem in anosmic patients. Furthermore, QoL and self‐esteem were correlated with depressive symptoms.

**Conclusion:**

As self‐esteem, QoL and depressive symptoms in anosmia interact with each other, we suggest that self‐esteem should be considered in the medical history, in order to provide a personalised intervention, adapted to the patient's needs.

## 1 Introduction

Olfactory dysfunction is a common disorder, affecting about 12% of general population, increasing with higher age.^1^ Interestingly, self‐reports are hardly reliable with respect to objectively measured olfactory function.^2^ Thus, smell disorders were often detected months after the actual onset. The effects of smell loss can be diverse. Affected patients are faced with a decreased ability to identify personal hazards, such as fire, gas or spoiled food.^3^ Furthermore, previous research suggests an impaired quality of life (QoL) and an increase in the risk of developing mental disorders (for review, see^4^). The wide‐spread consequences of smell loss do not only affect the patients themselves, they also cause a burden for the public and private health system.

Recent research observed effects of smell loss on everyday life even beyond obvious limitations with respect to personal hazards. Many affected patients suffer from reduced QoL, and they have a higher risk to develop depressive symptoms.^5^ Smeets et al.^5^ found that olfactory dysfunctions had substantial effects on QoL, especially related to situations in which the chemical senses play an important role. Therefore, the authors suggest that cognitive behavioural interventions may be provided as support for patients with severe smell loss. A study published by Shu et al.^6^ observed that olfactory loss was most challenging for younger patients with more severe olfactory disorders. Older patients, especially with longer disease duration, developed better coping strategies to reduce the influence of olfactory dysfunction on QoL. Previous research in the field of depression identified lower self‐esteem as a risk factor for developing depressive symptoms (for review, see^7^). Even though it has been shown that QoL was reduced in patients with olfactory dysfunction, little is known about the interplay of QoL, depressive symptoms and self‐esteem. However, these parameters are of particular interest, to satisfy the patient's needs and provide the most promising therapeutic intervention.

It has been established that olfactory dysfunction and depressive symptoms are associated in two ways. First, patients diagnosed with major depressive disorder often exhibit decreased olfactory function (for review see^8^). Second, patients with olfactory loss are more likely to develop depressive symptoms.^9^ Current estimates suggest that approximately one‐third of patients with smell disorders show at least mild depressive symptoms.^4^ A recent cohort‐controlled study in patients with chronic rhinosinusitis assumed that depressive symptoms were underdiagnosed in this patient group. The authors detected depressive symptoms, using the Beck Depression Inventory (BDI), in nearly one‐third of investigated patients.^10^

Although previous demographic investigations identified a correlation between smell disorders, a decrease in QoL, and a higher likelihood of developing depressive symptoms, little is known about the involvement of self‐esteem in these factors in patients with smell disorders. Motivation and committment to therapy is often a crucial factor for successful treatment. Particularly in long‐term interventions, such as olfactory training (for review, see^11^, ^12^), it is extremely important to maintain compliance. We, therefore, aimed to investigate self‐esteem, QoL and depressive symptoms in anosmic patients compared to healthy controls. Based on previous findings, we hypothesised a decreased self‐esteem in anosmic patients compared to healthy controls.

## 2 Materials and methods

### 2.1 Ethical considerations

The study was performed in accordance with the Declaration of Helsinki (1964), and the study protocol was approved by the Ethics Committee of the Medical University of Vienna. All subjects were informed about the aim of the study and gave written, informed consent prior to inclusion.

### 2.2 Subjects

Twenty‐two anosmic patients (12 females, 10 males) and 25 healthy subjects (15 females, 10 males) participated in this study. All subjects had no history of neurologic or psychiatric diseases, and no history of severe head trauma. Parts of this cohort had already participated in other studies of our research group.^13^, ^14^ Data on self‐esteem were available for only 17 anosmic patients (11 females, six males) and 12 healthy controls (five females, seven males). Healthy controls aged between 18 and 60 years were recruited via announcements at the Medical University of Vienna. A detailed description of the study sample is presented in Table 1.

**Table 1:** Descriptive statistics and results of olfactory performance measures and psychological questionnaires in the study sample

	Anosmic patients Mean (SD)	Healthy controls Mean (SD)
Number of participants (females/males)	22 (12/10)	25 (15/10)
Age (in years)	44.82 (11.57)	35.12 (11.42)
Disease duration (in years)	5.32 (6.26)	‐
TDI	12.11 (2.82)	35.42 (2.55)
Threshold	1.59 (1.00)	8.78 (1.79)
Discrimination	6.00 (2.02)	13.00 (1.50)
Identification	4.50 (1.92)	13.32 (1.91)
WHOQOL‐BREF^a^		
Physical health	82.55 (9.43)	87.36 (8.92)
Psychological	68.32 (15.23)	76.88 (9.54)
Social relationships	74.77 (18.53)	72.04 (20.75)
Environment	77.73 (10.79)	80.72 (8.24)
BDI	5.22 (4.36)	4.08 (3.94)
MSWS^b^		
Total self‐esteem	51.71 (35.12)	65.67 (25.51)
General self‐esteem	51.53 (33.06)	60.17 (29.68)
Body‐related self‐esteem	54.82 (30.29)	75.00 (19.25)

a Scores are presented as percentile ranks compared to a normative sample.

b Scores are presented as percentile ranks compared to a normative sample; for the MSWS, data from 17 anosmic patients and 12 healthy controls were available.

**Keypoints**

- Self‐esteem is an important factor in anosmic patients.
- Self‐esteem, quality of life and depressive systems act as highly interactive factors in anosmic patients.
- Self‐esteem should therefore be assessed in medical history.
- Self‐esteem may be important to provide a treatment adapted to the patient's needs.

### 2.3 Olfactory performance

Olfactory performance was assessed using the Sniffin’ Sticks test battery (Burghart Instruments, Wedel, Germany), comprising the three subtests: the odour detection threshold test; the odour discrimination test; and the odour identification test. A detailed description of the testing procedure is presented in Hummel et al.^15^ For the odour detection threshold, scores range from 1 to 16, and for the other two subtests, scores from 0 to 16 may be achieved. The results of all three subtests were summed to evaluate overall olfactory performance—the TDI (threshold‐detection‐identification) score, which can range from 1 to 48. Anosmia was defined by a TDI score of 17 or less, and normal olfactory performance was defined by a TDI score of at least 31.^16^

### 2.4 Behavioural data

Quality of life depicts the general well‐being of a subject. Subjective QoL was assessed using the German version of the WHOQOL‐BREF,^17^ a self‐reporting assessment. The WHOQOL‐BREF is a short version of the WHOQOL‐100, comprising 26 items, and covering four domains of QoL: physical health, psychological, social relationships and environment. The main advantage of this questionnaire is the quick and easy conduction. Furthermore, not only a general QoL will be assessed, but also information on different aspects will be provided in detail. Raw scores for each domain were transformed into percentile ranks according to normative data, as provided in Hawthorne et al.^18^

Depressive symptoms were evaluated using the German version of the Beck Depression Inventory II (BDI‐II^19^). This multiple‐choice self‐reporting questionnaire captures the severity of depressive symptoms. It consists of 21 items covering the most important symptoms of major depression disease, such as hopelessness, feelings of guilt or being punished as well as physical symptoms like fatigue and appetite loss. The BDI‐II is a well‐known inventory frequently used in psychiatry and psychology, not only in scientific research but also in clinical practice. Scores range from 0 to 63, and scores from 0 to 8 are defined as no depression, 9‐13 minimal depressive symptoms, 14‐19 mild depressive symptoms, 20‐28 moderate depressive symptoms and scores of 29 and higher reflect severe depressive symptoms.

Self‐esteem reflects a person's subjective evaluation of his or her own worth. It includes beliefs and emotional states about oneself. Self‐esteem was investigated using the Multidimensionale Selbstwertskala (MSWS^20^), a German questionnaire based on the Multidimensional Self‐Concept Scale (MSCS). The MSWS is a hierarchically structured self‐reporting assessment of different facets of self‐esteem, such as emotional self‐esteem, performance‐related self‐esteem, or physical attractiveness. The subscales are summed to two superordinate scales: general self‐esteem and body‐related self‐esteem. These two higher‐order scales are then added to a total self‐esteem score. The main advantage of this questionnaire is the covering of various aspects of self‐esteem, to provide more detailed information. The raw data from this questionnaire were transformed to percentile ranks, as provided in the test manual.

### 2.5 Statistical analysis

Statistical analysis was performed using the Statistical Package for the Social Sciences (SPSS, Chicago, IL, USA), version 20.0. For all test scores, mean and standard deviation were calculated. As all variables fulfilled the requirements for parametric testing, data were analysed using a one‐way multivariate analysis of variance (MANOVA). For group comparison of depressive symptoms, a two‐sample *t*‐test was performed. The alpha level for statistical testing was set to α=0.05. In addition to traditional statistical parameters, the Bayes factor (BF)^21^ will be reported for all univariate comparisons as well as for all correlations. The BF was calculated using the software JASP 0.8.0.1 (https://jasp-stats.org/). The BF is a relative likelihood ratio for the null hypothesis and the alternative hypothesis. High values represent a likelihood favouring the null hypothesis, low or negative values represent a likelihood favouring the alternative hypothesis.

## 3 Results

### 3.1 Quality of life

The QoL questionnaire (WHOQOL‐BREF) scores were not significantly correlated with age (physical health: *r*=.054, *P*=.720, BF_01_=5.166; psychological: *r*=−.246, *P*=.096, BF_01_=1.428; social relationships: *r*=−.085; *P*=.571, BF_01_=4.708; environment: *r*=−.043, *P*=.774, BF_01_=5.283) or gender (physical health: *r*=−.114, *P*=.447, BF_01_=4.151; psychological: *r*=−.202, *P*=.172, BF_01_=2.229; social relationships: *r*=−.006; *P*=.966, BF_01_=5.494; environment: *r*=−.026, *P*=.864, BF_01_=5.386).

**Figure 1:**
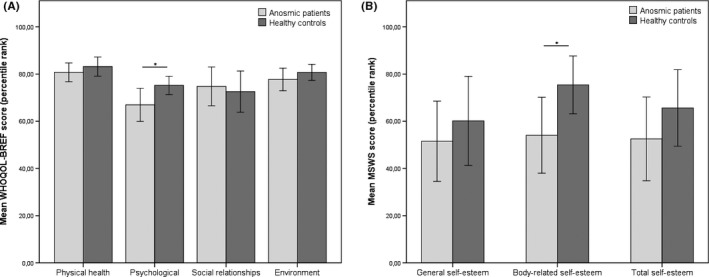
Mean percentile ranks of (A) WHOQOL‐BREF and (B) MSWS in patients with anosmia and healthy controls. Significant differences are marked with an asterisk

The four domains of the WHOQOL‐BREF were compared between anosmic patients and healthy controls using a one‐way MANOVA with the dependent variables physical health, psychological, social relationships and environment. Statistical analysis revealed a significant main effect of olfactory dysfunction on the combined variables (F(4,42)=2.782, *P*=.039, Wilks’ λ=0.791). In a next step, all dependent variables were analysed separately. A significant effect of olfactory dysfunction was observed in the psychological domain (F(1,45)=4.823, *P*=.033, ω_p_²=0.075; see Figure 1).

### 3.2 Depressive symptoms

No statistically significant difference in depressive symptoms was observed between anosmic patients and healthy controls (*t*(41)=0.898, *P*=.375, BF_01_=2.389). However, a significant negative correlation was obtained between the psychological domain of the WHOQOL‐BREF and the BDI scores (*r*=−.611, *P*<.001, BF_01_=0.001), as well as the body‐related self‐esteem and the BDI scores (*r*=−.428, *P*=.033, BF_01_=0.968) in the total study sample.

### 3.3 Self‐esteem

Self‐esteem (MSWS) scores were not significantly correlated with age (total self‐esteem: *r*=−.207, *P*=.280, BF_01_=2.485; general self‐esteem: *r*=−.195, *P*=.310, BF_01_=2.650; body‐related self‐esteem: *r*=−.247; *P*=.197, BF_01_=1.962) or gender (total self‐esteem: *r*=.099, *P*=.611, BF_01_=3.831; general self‐esteem: *r*=.060, *P*=.757, BF_01_=4.140; body‐related self‐esteem: *r*=.086; *P*=.659, BF_01_=3.949). General self‐esteem was significantly correlated with the psychological domain (*r*=.676, *P*<.001, BF_01_=0.002), with social relationships (*r*=.469, *P*=.010, BF_01_=0.188), and with environment (*r*=.495, *P*=.006, BF_01_=0.124) of the WHOQOL‐BREF. Body‐related self‐esteem was associated only with the psychological domain (*r*=.500, *P*=.006, BF_01_=0.144).

The one‐way MANOVA with the dependent variables total self‐esteem, general self‐esteem, and body‐related self‐esteem, revealed a significant main effect of olfactory dysfunction (F(3,25)=1.665,*P*=.200, Wilks’ λ=0.833) on the combined dependent variables. Detailed analysis, corrected for multiple testing using Bonferroni correction, revealed that olfactory dysfunction had a statistically significant effect on only one of the three subscales of the MSWS: body‐related self‐esteem (F(1,27)=4.391, *P*=.046, ω_p_²=0.110), with decreased scores for patients with olfactory dysfunction (see Figure 1).

## 4 Discussion

### 4.1 Synopsis and key findings

The main aim of the study was to investigate self‐esteem and QoL in anosmic patients compared to healthy controls. The results of this study revealed that anosmic patients experience a decreased QoL in the psychological domain, and significantly reduced body‐related self‐esteem. The medium effect size (ω_p_²=0.110) suggests that the reduced body‐related self‐esteem is not only statistically significant, but also clinically relevant. Although no statistically significant differences in depressive symptoms were observed between anosmic patients and healthy controls, the scores in the psychological domain were highly correlated with depressive symptoms.

### 4.2 Comparison with other studies

Low self‐esteem has been considered an important factor in major depression disorder for decades.^22^ Recent research discovered that low self‐esteem is an important risk factor for the development of depressive symptoms across the complete life span.^7^ This interaction was also found in patients with olfactory dysfunction who participated in the present study. Although no causal relationship can be derived from correlational analyses, the findings of our study are in line with the vulnerability model of depression (for review, see^23^), which assumes that low self‐esteem is a risk factor for depression.

Self‐esteem has already been identified as an important factor in self‐reported QoL.^24^ A recent study in breast cancer survivors reported self‐esteem as the strongest predictor of global QoL.^25^ It is assumed that higher self‐esteem has a positive effect on coping strategies,^26^ and on managing the stress in chronic or life‐threatening diseases.^27^ The results of the present study have shown that self‐esteem is highly associated with QoL, and with depressive symptoms in anosmic patients.

Previous studies have shown that specific olfactory performance training can induce a partial recovery of olfactory perception. Long‐term interventions, with a duration of approximately 18 weeks, such as olfactory training,^11^ require a high degree of motivation, as it is crucial to perform the training regularly over a time period of at least 3 months. As patients with comorbid depressive symptoms typically suffer from decreased energy and loss of interest, patients with depressive symptoms may profit less from this intervention than patients with less depressive symptoms. Furthermore, a recent study in patients with cardiovascular conditions reported a significant association between low self‐esteem and non‐compliance.^28^ The results of the present study have shown that QoL, self‐esteem, and depressive symptoms are highly interactive in patients with olfactory dysfunction. Therefore, these variables should be taken into account in patients with smell disorder, in order to provide a personalised intervention approach adapted to the individual needs of the patient. Future large‐scale, cohort‐controlled studies may discover the importance of treating any accompanying depressive symptoms and low self‐esteem along with therapy for an olfactory disorder, to strengthen the effect of the olfactory training.

### 4.3 Strengths and limitations of the study

This is the first study investigating the impact of self‐esteem on QoL and depressive symptoms in anosmic patients. Data of a homogeneous study sample of anosmic patients were acquired. However, there are still some limitations on this study. A potential limitation of this study is that healthy controls were significantly younger than patients with olfactory dysfunction. However, no significant correlations between age and the behavioural measures (WHOQOL‐BREF, BDI, and MSWS) were observed. Furthermore, data from the MSWS were available for only a part of the complete study sample. Another important issue is the application of correlation analyses in smaller samples. We therefore conducted a post‐hoc power analysis using the statistical program G*Power (http://www.gpower.hhu.de/), resulting in a power (1−β)=0.81 with a total sample size of n=47 and medium effect sizes of |ρ|=0.35. For the analysis of self‐esteem, the statistical power decreased to 0.60 because of the reduced sample size. However, despite this reduced statistical power, a statistically significant difference was obtained. The study sample was recruited for another investigation conducted by our study group.^14^ Only a part of the recruited participants agreed to fill in additional questionnaires. However, future studies on larger study samples with a broad variety of olfactory dysfunction are required to gain a deeper insight into the complex interaction of self‐esteem, QoL and depressive symptoms in patients with olfactory dysfunction. Moreover, future studies may also investigate individual life situations, such as occupation, education or family status in more detail.

## 5 Conclusion

The present study is the first investigating self‐esteem in patients with anosmia. The results have shown that self‐esteem plays a crucial role in these patients. We assume that self‐esteem and QoL are important factors in the risk for developing depressive symptoms in patients with smell disorder. We therefore suggest collecting data regarding self‐esteem and depressive symptoms in patients with olfactory dysfunction, in order to grain a deeper insight into the complex interaction of these parameters and develop individually adapted therapeutic interventions according to the needs of the patient.

## Author contributions

K. Kollndorfer and V. Schöpf designed the study. J.L. Reichert, B. Brückler and V. Hinterleitner acquired the data and supported data analysis. K. Kollndorfer analysed the data. K. Kollndorfer and V. Schöpf wrote the manuscript. All authors revised the article critically.

## Conflict of interest

None declared.
